# Genetic markers involved in neuroinflammation in Down syndrome: a systematic review

**DOI:** 10.1590/1980-5764-DN-2024-0251

**Published:** 2025-07-18

**Authors:** Marina Nascimento Silva, Mariana Dias Lula, Lucas Reis Felício, Bárbara Santos, Lívia Silva Nassif, Marcus Carvalho Borin, Juliana Alvares-Teodoro, Francisco de Assis Acurcio, Augusto Afonso Guerra

**Affiliations:** 1 Universidade Federal de Minas Gerais, Faculdade de Farmácia, Departamento de Farmácia Social, Belo Horizonte MG, Brazil.

**Keywords:** Down Syndrome, Neuroinflammatory Diseases, Alzheimer Disease, Genetic Markers, Genes, Complement System Proteins, Cytokines, Síndrome de Down, Doenças Neuroinflamatórias, Doença de Alzheimer, Marcadores Genéticos, Genes, Proteínas do Sistema Complemento, Citocinas

## Abstract

The immune system plays a fundamental role in protecting human body organs and tissues; however, when exacerbated, it can contribute to the pathology of various conditions. In the central nervous system, immune cell activation, or neuroinflammation, is a key factor in several neurodegenerative diseases. In Down syndrome (DS), the additional copy of chromosome 21 alters gene expression, potentially enhancing inflammatory processes such as neuroinflammation. Therefore, understanding the genetic factors influencing neuroinflammation in DS is essential for identifying biomarkers and therapeutic targets.

**Objective:**

Identify genetic markers involved in neuroinflammatory processes in individuals with DS.

**Methods:**

A comprehensive search was conducted in Medical Literature Analysis and Retrieval System Online (Medline) (United States National Library of Medicine [PubMed]), Embase, Cochrane Library, and Latin American and Caribbean Health Sciences Literature (LILACS) databases, and identified ten relevant studies. These studies assessed and compared gene expression between groups with and without DS associated with neuroinflammation.

**Results:**

Sixty–three genes and 42 genetic markers associated with neuroinflammation in DS were identified. These genes exhibited expression variations that alter inflammatory responses, suggesting a possible link to the progression of neurodegenerative diseases in this population.

**Conclusions:**

The findings highlight the role of neuroinflammation in neurodegenerative disorders in individuals with DS, especially Alzheimer’s disease. Some studies indicated that the triplicated genes SOD1, APP, S100B, TREM2, IFNR1, and IFNR2 are directly related to neuroinflammation. Additionally, elevated levels of pro-inflammatory cytokines, such as IL–1, IL–6, IL–10, IFNγ, and TNF-α, and complement proteins like C1q, C3, and C9 suggest an exacerbated activation of the immune response. However, the roles these genes may play in neurodegenerative diseases and in increasing or reducing neuroinflammation remain controversial.

## RESUMO

. O sistema imunológico é fundamental para a proteção dos órgãos e tecidos humanos; contudo, quando exacerbado, pode contribuir para a patologia de diversas condições. No sistema nervoso central, a ativação de células imunológicas, ou neuroinflamação, é um fator–chave em várias doenças neurodegenerativas. Na síndrome de Down (SD), a cópia adicional do cromossomo 21 altera a expressão gênica, intensificando processos inflamatórios como a neuroinflamação. Assim, entender os fatores genéticos associados à neuroinflamação na SD é essencial para identificar biomarcadores e alvos terapêuticos.

**Objetivo:**

Identificar marcadores genéticos envolvidos em processos neuroinflamatórios em indivíduos com SD.

**Métodos:**

Uma busca abrangente foi realizada nas bases de dados Sistema Online de Busca e Análise de Literatura Médica (MEDLINE) (Biblioteca Nacional de Medicina dos Estados Unidos [PubMed]), Embase, Cochrane Library e Literatura Latino–Americana e do Caribe em Ciências da Saúde (LILACS), incluindo dez estudos que compararam a expressão gênica entre grupos com e sem SD associados à neuroinflamação.

**Resultado:**

Foram identificados 63 genes e 42 marcadores genéticos associados à neuroinflamação na SD. Esses genes apresentaram variações de expressão que alteram as respostas inflamatórias, indicando uma possível relação com a progressão de doenças neurodegenerativas nessa população.

**Conclusões:**

Os achados destacam o papel da neuroinflamação em distúrbios neurodegenerativos em indivíduos com SD, especialmente na doença de Alzheimer. Alguns estudos demonstraram que genes triplicados, como SOD1, APP, S100B, TREM2, IFNR1 e IFNR2, estão diretamente relacionados à neuroinflamação. Níveis elevados de citocinas pró-inflamatórias, como IL-1, IL-6, IL-10, IFNγ e TNF-α, e de proteínas do complemento, como C1q, C3 e C9, sugerem uma ativação exacerbada da resposta imune. Contudo, o papel desses genes em doenças neurodegenerativas e neuroinflamação permanecem controversos.

## INTRODUCTION

 The immune system regulates and maintains bodily equilibrium by initiating cellular and molecular responses against foreign substances entering the body^1^ . These responses are categorized into two main defense lines: innate immunity, the initial and rapid response, and adaptive immunity, which provides a more specific and lasting defense^1 ,2^. Innate immunity includes physical barriers, such as the skin, immune cells like macrophages and dendritic cells, and effector molecules, including complement proteins and cytokines. Adaptive immunity, in contrast, is mediated by lymphocytes and antibody production^1 ,2^. While immunity generally serves to protect the body’s organs and tissues, excessive inflammatory responses can contribute to disease pathology, even when the primary cause of these conditions is not immunological^3^ . 

In the central nervous system (CNS), innate immune cells, such as microglia and astrocytes, play key roles in neuroinflammation by responding to damage and infections through specialized receptors that recognize pathogen-associated molecular patterns (PAMPs) or damage-associated molecular patterns (DAMPs) ^4^ . Activation of these cells can trigger chronic inflammation, contributing to the development of neurodegenerative diseases like Alzheimer’s disease (AD). In the context of AD, β-amyloid (Aβ) deposition in the brain activates microglia, which release pro-inflammatory cytokines, such as IL-1B, IL-6, and TNF-α, intensifying neuroinflammation ^5^ . Additionally, oxidative stress resulting from excessive mitochondrial free radical production also damages neurons, worsening the pathology. A though innate immunity is a primary contributor to AD pathology, some evidence suggests a subtler role for adaptive immunity, including alterations in T-lymphocyte levels observed in certain patients. Initially, there is an infiltration of T cells into brain regions proximal to activated microglia, such as the hippocampus. However, as AD progresses to dementia, there is an increase in microglia-mediated immune activity, accompanied by a reduction in T cell numbers ^6^ .

Microglia serve as resident macrophages in the CNS, acting as the first line of immune defense by continuously surveying the brain environment and responding swiftly to damage or pathogens through phagocytosis and the release of inflammatory cytokines^6 -8^. Astrocytes, on the other hand, provide essential structural and metabolic support to neurons, regulate the extracellular environment, participate in the blood-brain barrier, and help clear potentially neurotoxic waste, such Genetic markers in neuroinflammation. Silva MN, et al. as amyloid and tau proteins^9 ,10^. Together, microglia and astrocytes constitute the CNS’s primary immune components, responsible for pathogen clearance, removal of toxic residues, and maintaining synaptic homeostasis and neuronal plasticity^10 -13^ . 

 In Down syndrome (DS), both the innate and adaptive immune systems are generally compromised. Individuals with DS experience a higher incidence of infections and autoimmune diseases than the general population^ 14 ,15^. Additionally, studies have reported abnormal activation of microglia and astrocytes in individuals with DS, suggesting a predisposition toward an exaggerated neuroinflammatory response. This heightened inflammation likely contributes to neurodegeneration and the development of cognitive deficits and neurodegenerative diseases within this population^16 -18^. 

 Activated microglia release a range of inflammatory mediators, including cytokines and chemokines, which not only prolong inflammation but also disrupt neuron-to-neuron communication, impairing synaptic plasticity and signal transmission^16^ . This chronic activation is seen as a contributing factor to the cognitive and behavioral deficits associated with DS and is a common feature of neurodegenerative disorders. Microglial activation is observed in adolescents and young adults with DS and tends to intensify in adults with prominent AD pathology. In addition to microglia, dysregulated astrocyte activation in DS can disrupt their regulatory functions, further promoting neuroinflammation. The increased release of pro-inflammatory factors by these cells may heighten neurotoxicity and compromise the integrity of neuronal networks^19^ . 

 An increasing number of genes involved in neuroinflammation become upregulated in the aging brain, paralleling cognitive decline, as revealed by genome-wide expression studies^18 ,20-22^. Some genes located on chromosome 21, such as the APP (amyloid precursor protein) gene and interferon receptor genes, are linked to proteins that can induce neuroinflammation^19 ,23^. Overexpression of APP gene can lead to increased production of Aβ proteins, which are associated with microglial activation and chronic neuroinflammation. Aβ peptides may be recognized by microglial PAMPs, further activating phagocytic and inflammatory pathways^23^ . Enhanced phagocytic capacity enables microglia to internalize and degrade Aβ while triggering additional inflammatory pathways, such as the NF-κB pathway, which induces the expression of pro-inflammatory cytokines. Some of these cytokines, such as IL-1β, TNFα, and IL-6, are regulated by interferon receptor signaling and can, in turn, stimulate further glial activation, neuronal damage, and the upregulation of enzymes involved in generating pathogenic Aβ species, thus perpetuating a cycle of dysregulation. Moreover, the overexpression of interferon-related genes further amplifies the production of these cytokines, exacerbating neuroinflammation and contributing to disease progression^18 ,19 ,23^. 

 In this context, understanding the genetic factors that influence neuroinflammation in DS is crucial for developing new therapeutic approaches. Interventions targeting the modulation of the inflammatory response, such as anti-inflammatory or neuroprotective agents, hold the potential to enhance cognitive function and overall health in individuals with DS. Given this scenario, this systematic review aimed to identify key genetic markers associated with neuroinflammation in individuals with DS. 

## METHODS

 A systematic review was conducted using research from scientific publications indexed in online databases. This review followed the Preferred Reporting Items for Systematic Reviews and Meta-Analyses (PRISMA) 2020 guidelines and was registered in the International Prospective Register of Systematic Reviews (PROSPERO) under ID: CRD42024596020 

### Search strategy

 The research question, 'Which genetic markers are involved in neuroinflammatory processes and autoimmunity in individuals with Down syndrome?' guided the development of the research methodology for this review, as outlined in the Population, Exposure, Comparator, and Outcome (PECO) framework presented in Table 1. 

**Table 1:** Population, Exposure, Comparator, and Outcome framework defining the research question.

P (population)	Individuals with Down syndrome
E (exposure)	Neuroinflammation
C (control)	Absence of neuroinflammation
O (outcome)	Genetic markers involved in neuroinflammation

Abbreviation: PECO, Population, Exposure, Comparator, and Outcome.

### Study Selection

 A systematic literature search was conducted in the Medical Literature Analysis and Retrieval System Online (Medline) (United States National Library of Medicine [PubMed]), Embase, Cochrane Library, and Latin American and Caribbean Health Sciences Literature (LILACS) databases. Study selection was based on pre-established inclusion and exclusion criteria. The inclusion criteria encompassed studies involving human subjects with DS of any age. Specifically, observational studies analyzing clinical and genetic conditions related to neuroinflammation were included. 

 Studies that focused solely on genetic or molecular aspects of DS without a link to neuroinflammation, or those assessing inflammatory factors in DS without correlating them with genetic markers through gene expression, were excluded. Additionally, non-peer-reviewed studies (e.g., reports, opinions, conference abstracts) and studies using genotyping or gene expression analysis on post-mortem brain tissue were excluded. 

 Title and abstract screening was conducted using the Rayyan QCRI platform, with two independent reviewers screening in duplicate and blinded to each other’s decisions. To resolve conflicts, a third independent reviewer conducted an objective evaluation based strictly on the inclusion and exclusion criteria, without input from the initial reviewers, ensuring impartiality. The third reviewer’s decision was considered definitive for the article’s inclusion status. At the end of this process, selected articles were exported to a spreadsheet for further analysis, ensuring transparency and consistency throughout the review process. 

## RESULTS

### Data extraction

**Figure 1:**
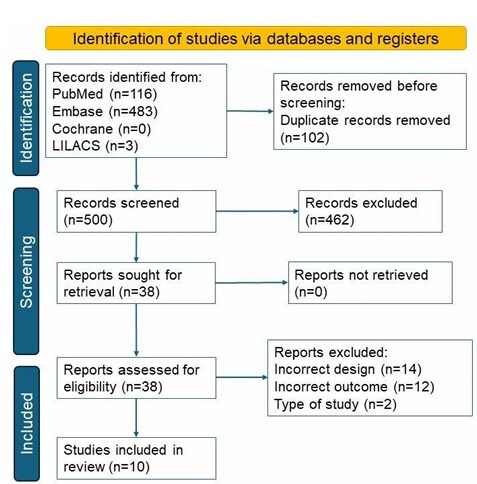
Preferred Reporting Items for Systematic Reviews and Meta-Analyses (PRISMA) flow diagram of article selection^24^ .

 The search identified a total of 602 scientific publications across the primary databases (PubMed, Embase, LILACS, and Cochrane). After removing 102 duplicate studies, 500 unique articles remained. Of these, 462 articles were excluded from the initial screening, and 38 articles remained. Following initial screening and the application of eligibility criteria, 12 articles were excluded for not addressing the research question, and 16 were excluded for not meeting the inclusion criteria. Ultimately, ten articles were included in the review. Figure 1 ^24^ illustrates the stages of study selection and the results obtained. 

 Among the included studies, nine were classified as cross-sectional and one as a cohort study. These studies evaluated the gene expression of genetic markers associated with neuroinflammation. The characteristics extracted from each study are summarized in Table 1. 

### Synthesis of results

 A total of 63 genes and 42 genetic markers associated with neuroinflammation in DS were identified. Additionally, the review identified cell types involved in neuroinflammation within this population.Table 2 ^25-34^ summarizes the findings of the ten included studies, providing details on the authors, year of publication, study design, sample population, participant characteristics, and identified genes. Supplementary Material I Table S1 presents a genetic map of the genes investigated across these studies. 

**Table 2:** Included articles and study characteristics.

Author	Study type	Objectives	Sample type	Methods	N (Participants)	Participant characteristics	Duration
Cairney et al.^25^	Cross-sectional	Investigate gene expression changes in hematopoietic stem cells (HSCs) and neural stem cells (NSCs) with aging	Bone marrow and human fetal tissue	Hematopoietic and neural stem cells: PolyA RT-PCR, cDNA: Microarray	200—1,000 cells	Children with DS without clinical hematological abnormalities undergoing cardiac surgery and agematched, hematologically healthy children undergoing similar significant surgery; individuals aged 60—80, free from any hematologic malignancy	n/a
Convertini et al.26.^26^	Cross-sectional	Investigate changes in the citrate pathway (oxidative stress) that may relate to certain phenotypic traits in DS	Blood	Blood sample collection (heparinized samples); lymphoblastoid cell culture; real-time PCR, SDS-PAGE, and Western blotting	20 total (2 groups: DS and control)	Children aged 3 to 5 years	n/a
Costa et al.^27^	Cross-sectional	Investigate the role of endothelial progenitor cells in DS	Blood (circulating endothelial progenitor cells from plasma)	Plasma samples; confocal immunofluorescence microscopy; quantitative RT-PCR	DS Group: 50; Euploid group: 30	Three age subgroups (young: 0—20 years; adults: 21—40 years; elderly: 41—60 years)	n/a
Donovan et al.^28^	Cross-sectional	Phenotype/genotype development in DS (gene expression pattern)	Blood	Blood sample collection	356 with DS and 146 controls	304 individuals with DS and 96 euploid controls; individuals with DS aged 6 months to 89 years	n/a
Jafarpour et al.^29^	Cohort	Investigate genes related to immune response regulation in individuals with DSRD	Not specified (retrospective cohort)	Not specified (retrospective cohort)	41 total	Individuals aged 10—30 years with DSRD	48 months
Mattos et al.^30^	Cross-sectional	Assess frequencies of interleukin 6 (IL-6) and IL-10 gene polymorphisms and serum levels of IL-6 and IL10 in healthy individuals with and without DS	Blood (serum/plasma)	PCR and Genotyping	200 (108 male, 95 female)	Children (age=4.3 years)	n/a
Raha-Chowdhur et al.^31^	Cross-sectional	Determine if genetic variants and inflammatory proteins are involved in hematopoiesis and cellular processes in DS compared to age-matched controls, particularly regarding neuroinflammation and accelerated aging	Blood	Blood smears and postmortem brain samples from individuals with AD and DS were analyzed by immunohistochemistry; RUNX1 mRNA expression analyzed by RT-PCR and in situ hybridization in mouse tissues	Blood samples: Control (n=50), DS (n=47)	Control group (ages 30—85 years); DS group (ages 32—70 years)	n/a
Silva et al.^32^	Cross-sectional	Investigate gene expression related to inflammatory processes in children with DS	Peripheral blood	RNA isolation from blood samples; complementary DNA synthesis (cDNA): quantitative real-time PCR (qPCR)	Control (n=6); DS (n=6)	Children without DS (control) and children with DS; DS group: Gender distribution: 4 male, 2 female; Age: 3.2 years (range: 2.1—6.6); Trisomy status: 5 had free chromosome 21 trisomy, one exhibited mosaicism with 90% of cells being trisomic	n/a
Trotta et al.^33^	Cross-sectional	Investigate humoral and cellular immunity parameters, RCAN1 gene expression, and cytokine production	Peripheral blood and tissue	Serology, immunophenotyping, quantitative gene expression of RCAN1, and cytokine production	DS (n=24) and control with intellectual disability without DS (n=21)	DS group (trisomy, age: 38±8.7 years); Control group (age: 43±8.3 years)	n/a
Veteleanu et al.^34^	Cross-sectional	Assess levels of complement system markers to identify DS-associated dysregulation and the influence of genotypes on these markers	Plasma, blood, and saliva	Genotyping	DS (n=71); control (n=46)	Gender, n (%): Female: DS: 25 (35.7%), Control: 29 (63%); Male: DS: 45 (64.3%), Control: 17 (37%); Age (Mean±SD): DS: 40.69 (13.22), Control: 37 (11.7)	n/a

 In general, the studies included in this review compared groups with and without DS to evaluate differences in the expression of genes associated with neuroinflammation. In some cases, comparisons were further subdivided among individuals with DS who had a diagnosis of AD or DSRD and those who did not. This approach enabled the identification of specific patterns of gene expression across the spectrum of aging and neurodegeneration. 

 Cairney et al.^25^ observed increased expression of the genes IL8, EGR1, Jag1, FOS, DAB2, SMAD2, and p21 in children with DS, showing an expression profile similar to that of older adults without DS. Convertini et al.^26^ reported overexpression of the genes ACLY and SREBP1 downregulation of CPT1 in individuals with DS. In another study, Costa et al.^27^ identified overexpression of IFNAR1, IFNAR2, SOD1, S100B, and APP in this population, with no differential expression observed for IL8 and DYRK1A. 

 Donovan et al.^28^ also reported overexpression of interferon receptor genes in individuals with DS. Jafarpour et al.^29^ investigated immunoregulatory genes linked to neuroinflammation that may contribute to DSRD. Regarding inflammatory cytokines, Mattos et al.^30^ found elevated levels of IL-10 in children with DS, without any influence from polymorphisms in the IL-10 gene. Raha-Chowdhur et al.^31^ identified lower serum levels of TREM2 in individuals without DS compared to those with DS. 

 Silva et al.^32^ reported differential expression of the genes BDKRB1 and LTA4H, both underexpressed in this group. Trotta et al.^33^ observed elevated levels of IFNγ, TNFα , and IL-10 in the DS group, with no difference in the expression of RCAN1. Finally, Veteleanu et al.^34^ found that complement proteins C1q, C3, and C9 were significantly elevated in individuals with DS. These results reinforce a distinct inflammatory profile in individuals with DS, with variations in gene expression and inflammatory cytokine levels among the compared groups. 

### Risk of bias

 The risk of bias assessment was conducted using the Joanna Briggs Institute (JBI) Critical Appraisal Tool, which evaluates study quality across domains such as participant selection, measurement consistency, management of confounding factors, and transparency in reporting. This tool facilitated a structured assessment of each study’s methodological rigor and potential biases, providing a comprehensive evaluation of the reliability and validity of the findings in this review. Specific information for each study is provided in the Supplementary Material II . 

 The evaluation of bias across the included studies highlighted several areas of concern. First, participant selection exhibited some variability, with a few studies lacking clear inclusion criteria or adequate control matching, thus increasing the risk of selection bias. Second, most studies were cross-sectional, limiting the ability to infer causality due to the absence of longitudinal data — a factor particularly relevant given the dynamic nature of neuroinflammation. Additionally, inconsistencies were noted in outcome measurement methods, especially concerning gene expression quantification, as varied techniques with differing sensitivities were employed. Some studies did not adequately address potential confounding factors, such as age or comorbidities, which could influence gene expression levels and neuroinflammatory markers. Although all studies provided ethical approvals, details regarding blinding and potential conflicts of interest were inconsistently reported, indicating the need for cautious interpretation of the findings. 

## DISCUSSION

 The findings of this review underscore the role of neuroinflammation in neurodegenerative disorders among individuals with DS, with a particular focus on genes exhibiting inflammatory properties. Additionally, the studies highlight the correlation between neuroinflammation and other concurrent processes involved in the pathology of neurodegenerative diseases, especially AD, which has a higher prevalence in individuals with DS^35,36^. To facilitate a structured discussion, the results have been categorized into specific themes that reflect the distinct mechanisms underlying neuroinflammation and neurodegeneration in DS. 

## Immune response, innate immunity and adaptive immunity

 Premature aging, including T cell deficiencies that compromise adaptive immunity, is a characteristic observed in individuals with DS. One hypothesis discussed in the literature is the genetic link between genes associated with stem cell aging, potentially leading to T cell deficiency. Although innate immunity appears to play a more significant role in neuroinflammation than adaptive immunity, dysfunctional T cells may lead to an inadequate immune response, allowing neuroinflammation to escalate. This may contribute to neurodegenerative processes, such as those seen in AD, which is more common in individuals with DS^ 37-39^. Pro-inflammatory cytokines, such as IL-8, produced by T cells, contribute to chronic neuroinflammation, creating a feedback loop that further impairs T cell function and exacerbates neuroinflammatory conditions in DS^37^ . 

 The studies also highlight the importance of innate immune cells, such as microglia, which display various reactive states throughout the lifespan of individuals with DS. Chronic microglial activation in DS may drive the progression of AD in this population. These findings are consistent with previous in vitro studies, which suggested that microglial activation may serve as a link between Aβ deposition and neuronal degeneration^40,41^. 

## Genes triplicated on chromosome 21

 Evidence from Costa et al.^27^ supports the hypothesis that the triplication of immune system-related genes encoded on chromosome 21, such as SOD1, APP, and S100B, is directly linked to neuroinflammation. However, the specific roles these genes may play in neurodegenerative diseases and their impact on the modulation of neuroinflammation remain controversial. 

 Various animal models with transgenic mice have demonstrated a link between the SOD1 gene and the manifestation of AD-like symptoms^ 42-44^. In transgenic mouse models, overexpression of the SOD1 gene has shown protective and antioxidant effects^45^ . A cohort study of adults with DS evaluated candidate genes for AD, finding the strongest genetic associations with Aβ40 levels in single nucleotide polymorphisms (SNPs) within the SOD1 gene^46^ . This gene encodes superoxide dismutase 1 (SOD1), a cytoplasmic and mitochondrial protein that binds copper and zinc, acting as a potent endogenous antioxidant by converting superoxide radicals into molecular oxygen and hydrogen peroxide^47,48^. However, other studies suggest that SOD1 overexpression in individuals with DS may lead to increased oxidative damage mediated by free radicals. Additionally, overexpression of this gene has been observed in degenerating neurons in the brains of individuals with DS^49,50^. 

 Regarding the S100B gene, the findings align with previous evidence from animal models, where overexpression of this gene correlates with AD pathology. This gene encodes the cytokine S100B, derived from astrocytes^46,51^. In individuals with DS, increased expression of S100B is frequently observed, particularly due to its location on chromosome 21, which is triplicated in this population. Studies suggest that elevated S100B levels may exert neurotoxic effects by stimulating the release of pro-inflammatory cytokines and activating glial cells, leading to chronic neuroinflammation and neuronal damage. Additionally, S100B is associated with the activation of oxidative stress-related pathways, which worsen the neuroinflammatory condition in individuals with DS, heightening their vulnerability to neurodegenerative diseases such as AD^51-53^. 

 Genome-wide association studies (GWAS) indicate that the TREM2 gene, involved in inflammation, is a significant risk factor for AD^54,55^. TREM2 is a receptor on the surface of microglial cells that detects pathological changes in the brain and initiates a protective response by allowing microglia to alter their gene expression pattern and become activated^56^ . Risk variants of TREM2 associated with AD, such as loss-of-function mutations, disrupt this process by preventing microglial activation or reducing ligand binding and signaling^56,57^. This results in microglia remaining in a homeostatic state even under disease conditions, which is significantly associated with an increased risk of developing AD^58,59^. Given that microglia in individuals with DS already exhibit an exaggerated inflammatory response, risk mutations in TREM2 could further aggravate this condition, preventing microglia from adopting a protective response and promoting the advancement of AD-related pathologies^60 ,61^. 

 Studies on the brains of AD patients show that microglia are attracted to Aβ, but this response is weakened by risk variants in TREM2 and is nearly absent with complete loss of TREM2 function, as seen in animal models. Furthermore, the absence of functional TREM2 amplifies amyloid deposition, suggesting that TREM2 plays a crucial role in the initial protective response against AD progression, activating even in the earliest stages of Aβ aggregate deposition, well before clinical symptoms appear^60,61^. 

 The findings of Trotta et al.^33^ and Mattos et al.^30^ indicate that pro-inflammatory cytokines, including IL-1, IL-6, IL-10, IFNγ , and TNF-α, are significantly elevated in individuals with DS compared to controls, reinforcing the hypothesis of a dysregulated immune system in this population. These results suggest that these cytokines may serve as potential biomarkers for the development of dementia associated with DS, with their plasma levels reflecting early neuroinflammation. The elevated expression of these cytokines may also be amplified by peripheral factors, such as chronic inflammatory diseases (e.g., periodontal disease), which contribute to the worsening of both systemic and cerebral inflammatory conditions^60^ . 

 The overexpression of the astrocyte-derived cytokine S100B, along with IL-1β, is identified as an early event in individuals with DS, establishing a feedback loop between neuroinflammation and neurodegeneration. The increased expression of the APP gene on chromosome 21 may lead to the accumulation of cleaved fragments, such as sAPPα , which in turn activate microglia and induce the production of IL-1β, thereby amplifying the inflammatory response in the central nervous system. These inflammatory mechanisms may directly influence the neuropathogenesis of both AD and DS, creating an environment conducive to the progression of Aβ- and tau-related pathologies^62^ . 

 Additionally, elevated plasma levels of proteins associated with the glial response, such as glial fibrillary acidic protein (GFAP), observed in prodromal stages of dementia and AD, underscore the importance of neuroinflammation as a precursor event in DS. GFAP, which is highly correlated with cortical atrophy and cerebral amyloid accumulation, has demonstrated strong diagnostic performance in distinguishing symptomatic from asymptomatic individuals and shows a significant increase in those progressing to dementia. Therefore, both inflammatory cytokines — such as IL-1β, IL-6, and TNF-α — and glial proteins like GFAP are essential for understanding the inflammatory processes underlying dementia in individuals with DS^63^ . 

 The overexpression of interferon receptor genes, such as IFNR1 and IFNR2, observed in individuals with DS contributes to an intensified inflammatory phenotype, enhancing immune response and neuroinflammation. These findings support the hypothesis that an exacerbated inflammatory response in individuals with DS plays a central role in dementia progression. This underscores the importance of continued research on inflammatory cytokines as potential therapeutic targets and prognostic biomarkers in managing dementia within this population. 

## Complement system dysregulation

 The findings of this review indicate that alterations in the complement system may contribute to a chronic inflammatory state in individuals with DS, including neuroinflammation, which may increase susceptibility to AD and other neurodegenerative diseases in this group. Elevated levels of complement proteins, such as C1q, C3, and C9, as observed by Veteleanu et al.^34^ , suggest an exaggerated activation of this system in individuals with DS. The complement system, a fundamental component of innate immunity, functions to recognize and attack pathogens and damaged cells, playing a crucial role in the immune response64. However, in the context of DS, this hyperactivation may contribute to a chronic neuroinflammatory state, as complement proteins can induce direct neuronal injury and heightened microglial activation^65^ . This condition may lead to brain tissue damage and foster a neuroinflammatory environment that correlates with accelerated cognitive decline and dementia development in individuals with DS^66,67^. The intensified inflammatory profile, characterized by increased regulation of complement components, may play a central role in the neurodegenerative pathophysiology of this population. 

## Immunoregulation and cognitive regression disorders

 The influence of immunoregulatory genes, as observed by Jafarpour et al.^29^ , suggests that a chronically activated immune response may be directly linked to the development of rapid cognitive decline syndrome (RCDS). This disorder, characterized by a rapid decline in cognitive and behavioral abilities, appears to be associated with inadequate immune regulation, which not only sustains an elevated inflammatory state but also exacerbates neural degeneration through microglial activation and continuous cytokine release^68^ . Immunoregulatory genes, such as those involved in responses to interferons and other cytokines, play a central role in maintaining immune homeostasis. When dysregulated, they can lead to immune hyper-responsiveness, amplifying the release of inflammatory mediators and facilitating the sustained activation of microglial cells in the brain — a factor already associated with neurodegeneration^69^ . This inflammatory profile may predispose individuals with DS to disorders such as DSRD, marked by a rapid decline in cognitive and behavioral abilities^68,70^. Hyperactive immune responses and neuroinflammation not only accelerate neurodegeneration but may also disrupt synaptic plasticity and neuronal circuit function, which are essential for cognition and behavior. 

## Lipid metabolism and neuroinflammation

 Another relevant aspect is the role of lipid metabolism and synthesis. Convertini et al.^26^ observed the overexpression of ATP-citrate lyase (ACLY) and sterol regulatory element-binding protein 1 (SREBP1) and the downregulation of carnitine palmitoyltransferase 1 (CPT1), indicating significant metabolic alterations with potential implications for neuroinflammation and neurodegeneration. The overexpression of ACLY and SREBP1 suggests an increase in lipogenesis, as ACLY plays a crucial role in generating cytoplasmic acetyl-CoA from citrate, a key precursor for fatty acid synthesis. Similarly, SREBP1 is a transcription factor that regulates genes involved in fatty acid and cholesterol biosynthesis, processes often upregulated under neuroinflammatory and neurodegenerative conditions. In contrast, the downregulation of CPT1 reduces fatty acid transport to mitochondria, limiting lipid oxidation and favoring intracellular lipid accumulation, which may contribute to oxidative stress and cellular dysfunction. 

 In individuals with DS, such changes in lipid metabolism may intensify neuroinflammatory processes, as intracellular lipid accumulation is associated with the activation of inflammatory pathways. This dysregulated lipid profile may also increase vulnerability of the nervous system to damage, as altered lipid metabolism directly influences neuronal homeostasis and cell membrane function. Furthermore, mitochondrial dysfunction resulting from reduced CPT1 activity leads to the accumulation of reactive oxygen species (ROS), promoting a pro-inflammatory and neurotoxic environment. These metabolic alterations and their connection to neuroinflammation offer a potential pathway through which lipid dysfunction may drive the progression of neurodegeneration in DS^26^ . 

## Methodological limitations and future directions

 This systematic review identified genetic markers associated with neuroinflammation in individuals with DS, but it also revealed certain methodological limitations in the included studies. The studies exhibited variability in participant characteristics, case definitions, control selection, gene expression quantification methods, and statistical analyses — factors that should be considered when interpreting the results, as they may affect gene expression findings. 

 Notably, all included studies collected samples for gene expression quantification at a single time point. Since neuroinflammation is a dynamic process influenced by external and internal factors, such as infections and neurodegenerative disease progression, the lack of longitudinal follow-up may limit understanding of the long-term impact of gene expression on neuroinflammation. 

 Future research should aim to explore genetic markers involved in pathological changes in glial cells in DS more comprehensively, focusing on their contribution to neuroinflammation over time in this population. 

 In conclusion, this systematic review underscores the significant role of neuroinflammation and its genetic components in the neurodegenerative processes observed in individuals with DS. Genes located on chromosome 21, such as SOD1, APP, and S100B, contribute to an inflammatory phenotype that heightens susceptibility to AD and accelerates cognitive decline in this population. Additionally, dysregulation in lipid metabolism, complement system activation, and chronic immune responses mediated by microglia and astrocytes are key factors in promoting neurodegeneration in DS. These findings highlight potential therapeutic targets and biomarkers that could inform strategies aimed at mitigating neuroinflammation and improving neurological outcomes in individuals with DS. 

## Data Availability

No new data were generated or analyzed in this study.

## Supplementary Materials

Supplementary Material I Table S1

Supplementary Material II
