# Supplementary material for: Genetic markers involved in neuroinflammation in Down syndrome: a systematic review
Source: Dement Neuropsychol. 2025 Jul 18;19(Suppl 1):e20240251. doi: 10.1590/1980-5764-DN-2024-0251 (PMC12306487; doi:10.1590/1980-5764-DN-2024-0251)
Supplement: Supplementary Material II [file 1980-5764-dn-19-s1-e20240251-suppl02.pdf]

Suplemmentary Material

Table s2.A. Search strategy MEDLINE via Pubmed

Pubmed – 23/08/2024		
#1	(("Neuroinflammatory Diseases"[MeSH Terms]) OR ("Neuroinflammatory Diseases"[Text Word] OR "Disease, Neuroinflammatory"[Text Word] OR "Neuroinflammatory Disease"[Text Word] OR "Neuroinflammatory Disorders"[Text Word] OR "Disorder, Neuroinflammatory"[Text Word] OR "Neuroinflammatory Disorder"[Text Word] OR "Neuroinflammation"[Text Word] OR "Neuroinflammations"[Text Word])) OR ((Inflammation[MeSH Terms]) OR (Inflammation[Text Word] OR Inflammations[Text Word] OR Innate Inflammatory Response[Text Word] OR Inflammatory Response, Innate[Text Word] OR Innate Inflammatory Responses[Text Word]))	1,096,898
#2	("Down Syndrome"[MeSH Terms]) OR ("Down Syndrome"[Text Word] OR "Syndrome, Down"[Text Word] OR "Down's Syndrome"[Text Word] OR "Downs Syndrome"[Text Word] OR "Syndrome, Down's"[Text Word] OR "Mongolism"[Text Word] OR "Trisomy 21"[Text Word] OR "Trisomy G"[Text Word] OR "47,XX,+21"[Text Word] OR "47,XY,+21"[Text Word] OR "Down Syndrome, Partial Trisomy 21"[Text Word] OR "Partial Trisomy 21 Down Syndrome"[Text Word] OR "Trisomy 21, Meiotic Nondisjunction"[Text Word] OR "Trisomy 21, Mitotic Nondisjunction"[Text Word])	36,424
#3	("Genes"[MeSH Terms]) OR ("Genes"[Text Word] OR "Gene"[Text Word] OR "Genetic Materials"[Text Word] OR "Genetic Material"[Text Word] OR "Material, Genetic"[Text Word] OR "Materials, Genetic"[Text Word] OR "Cistron"[Text Word] OR "Cistrons"[Text Word])	
#4	(((("Neuroinflammatory Diseases"[MeSH Terms]) OR ("Neuroinflammatory Diseases"[Text Word] OR "Disease, Neuroinflammatory"[Text Word] OR "Neuroinflammatory Disease"[Text Word] OR "Neuroinflammatory Disorders"[Text Word] OR "Disorder, Neuroinflammatory"[Text Word] OR "Neuroinflammatory Disorder"[Text Word] OR "Neuroinflammation"[Text Word] OR "Neuroinflammations"[Text Word])) OR ((Inflammation[MeSH Terms]) OR (Inflammation[Text Word] OR Inflammations[Text Word] OR Innate Inflammatory Response[Text Word] OR Inflammatory Response, Innate[Text Word] OR Innate Inflammatory Responses[Text Word]))) AND (("Down Syndrome"[MeSH Terms]) OR ("Down Syndrome"[Text Word] OR "Syndrome, Down"[Text Word] OR "Down's Syndrome"[Text Word] OR "Downs Syndrome"[Text Word] OR "Syndro me, Down's"[Text Word] OR	3,424,015
**#5**	**(((("Neuroinflammatory Diseases"[MeSH Terms]) OR ("Neuroinflammatory Diseases"[Text Word] OR "Disease, Neuroinflammatory"[Text Word] OR "Neuroinflammatory Disease"[Text Word] OR "Neuroinflammatory Disorders"[Text Word] OR "Disorder, Neuroinflammatory"[Text Word] OR "Neuroinflammatory Disorder"[Text Word] OR "Neuroinflammation"[Text Word] OR "Neuroinflammations"[Text Word])) OR ((Inflammation[MeSH Terms]) OR (Inflammation[Text Word] OR Inflammations[Text Word] OR Innate Inflammatory Response[Text Word] OR Inflammatory Response, Innate[Text Word] OR Inn ate Inflammatory Responses[Text Word]))) AND (("Down Syndrome"[MeSH Terms]) OR ("Down Syndrome"[Text Word] OR "Syndrome, Down"[Text Word] OR "Down's Syndrome"[Text Word] OR "Downs Syndrome"[Text Word] OR "Syndrome, Down's"[Text Word] OR "Mongolism"[Text Word] OR "Trisomy 21"[Text Word] OR "Trisomy G"[Text Word] OR "47,XX,+21"[Text Word] OR "47,XY,+21"[Text Word] OR "Down Syndrome, Partial Trisomy 21"[Text Word] OR "Partial Trisomy 21 Down Syndrome"[Text Word] OR "Trisomy 21, Meiotic Nondisjunction"[Text Word] OR "Trisomy 21, Mitotic Nondisjunction"[Text Word]))) AND (("Genes"[MeSH Terms]) OR ("Genes"[Text Word] OR "Gene"[Text Word] OR "Genetic Materials"[Text Word] OR "Genetic Material"[Text Word] OR "Material, Genetic"[Text Word] OR "Materials, Genetic"[Text Word] OR "Cistron"[Text Word] OR "Cistrons"[Text Word]))**	**116**

Table s2.B Search strategy Embase

**Embase – 23/08/2024**		
#1	'nervous system inflammation'/syn OR 'nervous system inflammation'	406,294
#2	'inflammation'/syn OR 'inflammation'	5,006,168
#3	#1 OR #2	5,019,382
#4	'down syndrome'/syn OR 'down syndrome'	47,462
#5	'gene'/syn OR 'gene'	5,019,428
#6	#3 AND #4 AND #5	701
**#7**	**#3 AND #4 AND #5**	**¨483**

¨ Filtro: Artigos e revisões

Table s2.C Search strategy Cochrane

**Cochrane – 23/08/2024**		
#1	MeSH descriptor: [Neuroinflammatory Diseases] explode all trees	2330
#2	MeSH descriptor: [Inflammation] explode all trees	16560
#3	#1 OR #2	18795
#4	MeSH descriptor: [Down Syndrome] explode all trees	541
#5	MeSH descriptor: [Genes] explode all trees	2522
**#6**	**#3 AND #4 AND #5**	**0**

Table s2.D Search strategy Lilacs

**Lilacs – 23/08/2024**	
#1	(“Síndrome de Down” or “Down Syndrome” or “Síndrome de Down” or “Syndrome de Down” or “Trissomia do 21” or “Trissomia do Cromossomo 21”)
#2	(“Doenças Neuroinflamatórias” or “Neuroinflammatory Diseases” or “Enfermedades Neuroinflamatorias” or “Maladies neuro-inflammatoires” or “Distúrbios Neuroinflamatórios” or “Neuroinflamação” or “Neuroinflamações” or “Transtornos Neuroinflamatórios” or “Inflamação” or “Resposta Inflamatória Inata” or “Inflammation” or “Inflamación” or“Inflammation”)
#3	(“Genes”)
#4	((“síndrome de down” OR “down syndrome” OR “síndrome de down” OR “syndrome de down” OR “trissomia do 21” OR “trissomia do cromossomo 21”)) AND ((“doenças neuroinflamatórias” OR “neuroinflammatory diseases” OR “enfermedades neuroinflamatorias” OR “maladies neuro-inflammatoires” OR “distúrbios neuroinflamatórios” OR “neuroinflamação” OR “neuroinflamações” OR “transtornos neuroinflamatórios” OR “inflamação” OR “resposta inflamatória inata” OR “inflammation” OR “inflamación” or“inflammation”)) AND ((“genes”))
**3 results**	

Supplementary Material 2

Table s3. Excluded articles

Araya et al., 2019	Incorrect outcome
Cheon et al., 2001	Incorrect outcome
Day et al., 2016	Incorrect design
Dogliotti et al., 2010	Incorrect outcome
Doran et al., 2016	Type of study
Eun et al., 2008	Incorrect design
Fagan et al., 2021	Incorrect outcome
Guo et al. 2023	Incorrect outcome
Henson, 2020	Incorrect outcome
Hesser et al.,	Incorrect design
Huang et al., 2023	Incorrect outcome
Huggard, 2021	Incorrect outcome
Ko et al., 2014	Incorrect design
Lee et al., 2012	Incorrect design
Lee et al., 2009	Incorrect design
Li et al., 2012	Incorrect design
Lu et al., 2011	Incorrect design
Mahernia et al., 2020	Incorrect design
Martin et al., 2014	Incorrect design
Mehta et al., 2007	Incorrect outcome
Minami et al., 2006	Incorrect outcome
Rafii et al., 2021	Incorrect design
Raha-Chowdhury, 2018	Incorrect design
Raha-Chowdhury, 2019	Incorrect design
Startin et al., 2019	Incorrect outcome
Waugh et al., 2019	Incorrect outcome
Wilcock et al., 2015	Incorrect design
Zhao et al., 2017	Type of study

CHECKLIST FOR ANALYTICAL CROSS SECTIONAL STUDIES

Critical Appraisal tools for use in JBI Systematic Reviews

INTRODUCTION

JBI is an JBI is an international research organisation based in the Faculty of Health and Medical Sciences at the University of Adelaide, South Australia. JBI develops and delivers unique evidence-based information, software, education and training designed to improve healthcare practice and health outcomes. With over 70 Collaborating Entities, servicing over 90 countries, JBI is a recognised global leader in evidence-based healthcare.

JBI Systematic Reviews

The core of evidence synthesis is the systematic review of literature of a particular intervention, condition or issue. The systematic review is essentially an analysis of the available literature (that is, evidence) and a judgment of the effectiveness or otherwise of a practice, involving a series of complex steps. JBI takes a particular view on what counts as evidence and the methods utilised to synthesise those different types of evidence. In line with this broader view of evidence, JBI has developed theories, methodologies and rigorous processes for the critical appraisal and synthesis of these diverse forms of evidence in order to aid in clinical decision-making in healthcare. There now exists JBI guidance for conducting reviews of effectiveness research, qualitative research, prevalence/incidence, etiology/risk, economic evaluations, text/opinion, diagnostic test accuracy, mixed-methods, umbrella reviews and scoping reviews. Further information regarding JBI systematic reviews can be found in the JBI Evidence Synthesis Manual.

JBI Critical Appraisal Tools

All systematic reviews incorporate a process of critique or appraisal of the research evidence. The purpose of this appraisal is to assess the methodological quality of a study and to determine the extent to which a study has addressed the possibility of bias in its design, conduct and analysis. All papers selected for inclusion in the systematic review (that is – those that meet the inclusion criteria described in the protocol) need to be subjected to rigorous appraisal by two critical appraisers. The results of this appraisal can then be used to inform synthesis and interpretation of the results of the study. JBI Critical appraisal tools have been developed by the JBI and collaborators and approved by the JBI Scientific Committee following extensive peer review. Although designed for use in systematic reviews, JBI critical appraisal tools can also be used when creating Critically Appraised Topics (CAT), in journal clubs and as an educational tool.

© JBI, 2020. All rights reserved. JBI grants use of these tools for research purposes only. All other enquiries should be sent to jbisynthesis@adelaide.edu.au.

JBI CRITICAL APPRAISAL CHECKLIST FOR ANALYTICAL CROSS SECTIONAL STUDIES

Reviewer Marina Nascimento Silva Date 2024/10/25

Author Cairney Year 2009 Record Number________

	Yes	No	Unclear	Not applicable
1. Were the criteria for inclusion in the sample clearly defined?	☐	☐	X	☐
2. Were the study subjects and the setting described in detail?	X	☐	☐	☐
3. Was the exposure measured in a valid and reliable way?	X	☐	☐	☐
4. Were objective, standard criteria used for measurement of the condition?	☐	☐	X	☐
5. Were confounding factors identified?	☐	☐	X	☐
6. Were strategies to deal with confounding factors stated?	☐	☐	X	☐
7. Were the outcomes measured in a valid and reliable way?	X	☐	☐	☐
8. Was appropriate statistical analysis used?	☐	☐	X	☐

Overall appraisal: Include X Exclude ☐ Seek further info ☐

Comments (Including reason for exclusion)

© JBI, 2020. All rights reserved. JBI grants use of these tools for research purposes only. All other enquiries should be sent to jbisynthesis@adelaide.edu.au.

JBI CRITICAL APPRAISAL CHECKLIST FOR ANALYTICAL CROSS SECTIONAL STUDIES

Reviewer Marina Nascimento Silva Date 2024/10/25

Author Convertini Year 2016 Record Number__________

	Yes	No	Unclear	Not applicable
9. Were the criteria for inclusion in the sample clearly defined?	☐	☐	X	☐
10. Were the study subjects and the setting described in detail?	☐	☐	X	☐
11. Was the exposure measured in a valid and reliable way?	X	☐	☐	☐
12. Were objective, standard criteria used for measurement of the condition?	☐	☐	X	☐
13. Were confounding factors identified?	X	☐	☐	☐
14. Were strategies to deal with confounding factors stated?	☐	☐	X	☐
15. Were the outcomes measured in a valid and reliable way?	X	☐	☐	☐
16. Was appropriate statistical analysis used?	X	☐	☐	☐

Overall appraisal: Include X Exclude ☐ Seek further info ☐

Comments (Including reason for exclusion)

© JBI, 2020. All rights reserved. JBI grants use of these tools for research purposes only. All other enquiries should be sent to jbisynthesis@adelaide.edu.au.

JBI CRITICAL APPRAISAL CHECKLIST FOR ANALYTICAL CROSS SECTIONAL STUDIES

Reviewer Marina Nascimento Silva Date 2024/10/25

Author Costa Year 2010 Record Number_________

	Yes	No	Unclear	Not applicable
17. Were the criteria for inclusion in the sample clearly defined?	☐	☐	X	☐
18. Were the study subjects and the setting described in detail?	X	☐	☐	☐
19. Was the exposure measured in a valid and reliable way?	X	☐	☐	☐
20. Were objective, standard criteria used for measurement of the condition?	X	☐	☐	☐
21. Were confounding factors identified?	X	☐	☐	☐
22. Were strategies to deal with confounding factors stated?	☐	☐	X	☐
23. Were the outcomes measured in a valid and reliable way?	X	☐	☐	☐
24. Was appropriate statistical analysis used?	X	☐	☐	☐

Overall appraisal: Include X Exclude ☐ Seek further info ☐

Comments (Including reason for exclusion)

Reviewer Marina Nascimento Silva Date 2024/10/25

© JBI, 2020. All rights reserved. JBI grants use of these tools for research purposes only. All other enquiries should be sent to jbisynthesis@adelaide.edu.au.

JBI CRITICAL APPRAISAL CHECKLIST FOR ANALYTICAL CROSS SECTIONAL STUDIES

Reviewer Marina Nascimento Silva Date 2024/10/25

Author Donavan Year 2024 Record Number__________

	Yes	No	Unclear	Not applicable
25. Were the criteria for inclusion in the sample clearly defined?	☐	☐	X	☐
26. Were the study subjects and the setting described in detail?	☐	☐	X	☐
27. Was the exposure measured in a valid and reliable way?	X	☐	☐	☐
28. Were objective, standard criteria used for measurement of the condition?	☐	☐	X	☐
29. Were confounding factors identified?	X	☐	☐	☐
30. Were strategies to deal with confounding factors stated?	☐	☐	X	☐
31. Were the outcomes measured in a valid and reliable way?	X	☐	☐	☐
32. Was appropriate statistical analysis used?	X	☐	☐	☐

Overall appraisal: Include X Exclude ☐ Seek further info ☐

Comments (Including reason for exclusion)

© JBI, 2020. All rights reserved. JBI grants use of these tools for research purposes only. All other enquiries should be sent to jbisynthesis@adelaide.edu.au.

JBI CRITICAL APPRAISAL CHECKLIST FOR ANALYTICAL CROSS SECTIONAL STUDIES

Reviewer Marina Nascimento Silva Date 2024/10/25

Author Jafarpour Year 2024 Record Number__________

	Yes	No	Unclear	Not applicable
33. Were the criteria for inclusion in the sample clearly defined?	X	☐	☐	☐
34. Were the study subjects and the setting described in detail?	X	☐	☐	☐
35. Was the exposure measured in a valid and reliable way?	X	☐	☐	☐
36. Were objective, standard criteria used for measurement of the condition?	X	☐	☐	☐
37. Were confounding factors identified?	☐	☐	X	☐
38. Were strategies to deal with confounding factors stated?	☐	☐	X	☐
39. Were the outcomes measured in a valid and reliable way?	X	☐	☐	☐
40. Was appropriate statistical analysis used?	X	☐	X	☐

Overall appraisal: Include X Exclude ☐ Seek further info ☐

Comments (Including reason for exclusion)

© JBI, 2020. All rights reserved. JBI grants use of these tools for research purposes only. All other enquiries should be sent to jbisynthesis@adelaide.edu.au.

JBI CRITICAL APPRAISAL CHECKLIST FOR ANALYTICAL CROSS SECTIONAL STUDIES

Reviewer Marina Nascimento Silva Date 2024/10/25

Author Mattos Year 2018 Record Number__________

	Yes	No	Unclear	Not applicable
41. Were the criteria for inclusion in the sample clearly defined?	☐	☐	X	☐
42. Were the study subjects and the setting described in detail?	☐	X	☐	☐
43. Was the exposure measured in a valid and reliable way?	X	☐	☐	☐
44. Were objective, standard criteria used for measurement of the condition?	☐	☐	X	☐
45. Were confounding factors identified?	X	☐	☐	☐
46. Were strategies to deal with confounding factors stated?	☐	☐	X	☐
47. Were the outcomes measured in a valid and reliable way?	X	☐	☐	☐
48. Was appropriate statistical analysis used?	X	☐	☐	☐

Overall appraisal: Include X Exclude ☐ Seek further info ☐

Comments (Including reason for exclusion)

© JBI, 2020. All rights reserved. JBI grants use of these tools for research purposes only. All other enquiries should be sent to jbisynthesis@adelaide.edu.au.

JBI CRITICAL APPRAISAL CHECKLIST FOR ANALYTICAL CROSS SECTIONAL STUDIES

Reviewer Marina Nascimento Silva Date 2024/10/25

Author Raha-Chowdhur Year 2021 Record Number_________

	Yes	No	Unclear	Not applicable
49. Were the criteria for inclusion in the sample clearly defined?	☐	☐	X	☐
50. Were the study subjects and the setting described in detail?	☐	X	☐	☐
51. Was the exposure measured in a valid and reliable way?	X	☐	☐	☐
52. Were objective, standard criteria used for measurement of the condition?	☐	☐	X	☐
53. Were confounding factors identified?	X	☐	☐	☐
54. Were strategies to deal with confounding factors stated?	☐	☐	X	☐
55. Were the outcomes measured in a valid and reliable way?	X	☐	☐	☐
56. Was appropriate statistical analysis used?	X	☐	☐	☐

Overall appraisal: Include X Exclude ☐ Seek further info ☐

Comments (Including reason for exclusion)

© JBI, 2020. All rights reserved. JBI grants use of these tools for research purposes only. All other enquiries should be sent to jbisynthesis@adelaide.edu.au.

JBI CRITICAL APPRAISAL CHECKLIST FOR ANALYTICAL CROSS SECTIONAL STUDIES

Reviewer Marina Nascimento Silva Date 2024/10/25

Author Silva Year 2016 Record Number_________

	Yes	No	Unclear	Not applicable
57. Were the criteria for inclusion in the sample clearly defined?	X	☐	☐	☐
58. Were the study subjects and the setting described in detail?	X	☐	☐	☐
59. Was the exposure measured in a valid and reliable way?	X	☐	☐	☐
60. Were objective, standard criteria used for measurement of the condition?	X	☐	☐	☐
61. Were confounding factors identified?	X	☐	☐	☐
62. Were strategies to deal with confounding factors stated?	☐	☐	X	☐
63. Were the outcomes measured in a valid and reliable way?	X	☐	☐	☐
64. Was appropriate statistical analysis used?	X	☐	☐	☐

Overall appraisal: Include X Exclude ☐ Seek further info ☐

Comments (Including reason for exclusion)

© JBI, 2020. All rights reserved. JBI grants use of these tools for research purposes only. All other enquiries should be sent to jbisynthesis@adelaide.edu.au.

JBI CRITICAL APPRAISAL CHECKLIST FOR ANALYTICAL CROSS SECTIONAL STUDIES

Reviewer Marina Nascimento Silva Date 2024/10/25

Author Trotta Year 2011 Record Number__________

	Yes	No	Unclear	Not applicable
65. Were the criteria for inclusion in the sample clearly defined?	☐	☐	X	☐
66. Were the study subjects and the setting described in detail?	X	☐	☐	☐
67. Was the exposure measured in a valid and reliable way?	X	☐	☐	☐
68. Were objective, standard criteria used for measurement of the condition?	☐	☐	X	☐
69. Were confounding factors identified?	☐	☐	X	☐
70. Were strategies to deal with confounding factors stated?	☐	☐	X	☐
71. Were the outcomes measured in a valid and reliable way?	X	☐	☐	☐
72. Was appropriate statistical analysis used?	☐	☐	X	☐

Overall appraisal: Include X Exclude ☐ Seek further info ☐

Comments (Including reason for exclusion)

© JBI, 2020. All rights reserved. JBI grants use of these tools for research purposes only. All other enquiries should be sent to jbisynthesis@adelaide.edu.au.

JBI CRITICAL APPRAISAL CHECKLIST FOR ANALYTICAL CROSS SECTIONAL STUDIES

Reviewer Marina Nascimento Silva Date 2024/10/25

Author Veteleanu Year 2022 Record Number__________

	Yes	No	Unclear	Not applicable
73. Were the criteria for inclusion in the sample clearly defined?	☐	☐	X	☐
74. Were the study subjects and the setting described in detail?	X	☐	☐	☐
75. Was the exposure measured in a valid and reliable way?	X	☐	☐	☐
76. Were objective, standard criteria used for measurement of the condition?	☐	☐	X	☐
77. Were confounding factors identified?	☐	☐	X	☐
78. Were strategies to deal with confounding factors stated?	☐	☐	X	☐
79. Were the outcomes measured in a valid and reliable way?	X	☐	☐	☐
80. Was appropriate statistical analysis used?	☐	☐	X	☐

Overall appraisal: Include X Exclude ☐ Seek further info ☐

Comments (Including reason for exclusion)

© JBI, 2020. All rights reserved. JBI grants use of these tools for research purposes only. All other enquiries should be sent to jbisynthesis@adelaide.edu.au.

EXPLANATION OF ANALYTICAL CROSS SECTIONAL STUDIES CRITICAL APPRAISAL

How to cite: Moola S, Munn Z, Tufanaru C, Aromataris E, Sears K, Sfetcu R, Currie M, Qureshi R, Mattis P, Lisy K, Mu P-F. Chapter 7: Systematic reviews of etiology and risk . In: Aromataris E, Munn Z (Editors). *JBI Manual for Evidence Synthesis.* JBI, 2020. Available from https://synthesismanual.jbi.global

Analytical cross sectional studies Critical Appraisal Tool

Answers: Yes, No, Unclear or Not/Applicable

1. Were the criteria for inclusion in the sample clearly defined?

The authors should provide clear inclusion and exclusion criteria that they developed prior to recruitment of the study participants. The inclusion/exclusion criteria should be specified (e.g., risk, stage of disease progression) with sufficient detail and all the necessary information critical to the study.

2. Were the study subjects and the setting described in detail?

The study sample should be described in sufficient detail so that other researchers can determine if it is comparable to the population of interest to them. The authors should provide a clear description of the population from which the study participants were selected or recruited, including demographics, location, and time period.

3. Was the exposure measured in a valid and reliable way?

The study should clearly describe the method of measurement of exposure. Assessing validity requires that a 'gold standard' is available to which the measure can be compared. The validity of exposure measurement usually relates to whether a current measure is appropriate or whether a measure of past exposure is needed.

Reliability refers to the processes included in an epidemiological study to check repeatability of measurements of the exposures. These usually include intra-observer reliability and inter-observer reliability.

4. Were objective, standard criteria used for measurement of the condition?

It is useful to determine if patients were included in the study based on either a specified diagnosis or definition. This is more likely to decrease the risk of bias. Characteristics are another useful approach to matching groups, and studies that did not use specified diagnostic methods or definitions should provide evidence on matching by key characteristics

5. Were confounding factors identified?

Confounding has occurred where the estimated intervention exposure effect is biased by the presence of some difference between the comparison groups (apart from the exposure investigated/of interest). Typical confounders include baseline characteristics, prognostic factors, or concomitant exposures (e.g. smoking). A confounder is a difference between the comparison groups and it influences the direction of the study results. A high quality study at the level of cohort design will identify the potential confounders and measure them (where possible). This is difficult for studies where behavioral, attitudinal or lifestyle factors may impact on the results.

6. Were strategies to deal with confounding factors stated?

Strategies to deal with effects of confounding factors may be dealt within the study design or in data analysis. By matching or stratifying sampling of participants, effects of confounding factors can be adjusted

© JBI, 2020. All rights reserved. JBI grants use of these tools for research purposes only. All other enquiries should be sent to jbisynthesis@adelaide.edu.au.

for. When dealing with adjustment in data analysis, assess the statistics used in the study. Most will be some form of multivariate regression analysis to account for the confounding factors measured.

7. Were the outcomes measured in a valid and reliable way?

Read the methods section of the paper. If for e.g. lung cancer is assessed based on existing definitions or diagnostic criteria, then the answer to this question is likely to be yes. If lung cancer is assessed using observer reported, or self-reported scales, the risk of over- or under-reporting is increased, and objectivity is compromised. Importantly, determine if the measurement tools used were validated instruments as this has a significant impact on outcome assessment validity.

Having established the objectivity of the outcome measurement (e.g. lung cancer) instrument, it's important to establish how the measurement was conducted. Were those involved in collecting data trained or educated in the use of the instrument/s? (e.g. radiographers). If there was more than one data collector, were they similar in terms of level of education, clinical or research experience, or level of responsibility in the piece of research being appraised?

8. Was appropriate statistical analysis used?

As with any consideration of statistical analysis, consideration should be given to whether there was a more appropriate alternate statistical method that could have been used. The methods section should be detailed enough for reviewers to identify which analytical techniques were used (in particular, regression or stratification) and how specific confounders were measured.

For studies utilizing regression analysis, it is useful to identify if the study identified which variables were included and how they related to the outcome. If stratification was the analytical approach used, were the strata of analysis defined by the specified variables? Additionally, it is also important to assess the appropriateness of the analytical strategy in terms of the assumptions associated with the approach as differing methods of analysis are based on differing assumptions about the data and how it will respond.

© JBI, 2020. All rights reserved. JBI grants use of these tools for research purposes only. All other enquiries should be sent to jbisynthesis@adelaide.edu.au.
